# Inhibiting Intestinal Krüppel-Like Factor 5 Impairs the Beneficial Role of Renal Denervation in Gut Microbiota in Rats with Heart Failure

**DOI:** 10.1128/spectrum.02183-22

**Published:** 2022-09-22

**Authors:** Zhiqin Guo, Fuyan Chen, Yufeng Chen, Chao Liu, Shaonan Li, Pingan Chen

**Affiliations:** a Department of Cardiology, the Second Affiliated Hospital, School of Medicine, South China University of Technology, Guangzhou, China; b Department of electrocardiogram, Guangzhou First People’s Hospital, School of Medicine, South China University of Technology, Guangzhou, China; Tainan Hospital, Department of Health, Executive Yuan

**Keywords:** gut microbiota, heart failure, Krüppel-like factor 5, renal denervation

## Abstract

Krüppel-like factor 5 (KLF5) is critical in maintaining intestinal barrier function, and renal denervation (RDN) mitigates gut microbiota aberrations in rats with heart failure (HF). It is unclear whether intestinal KLF5 can be regulated by RDN and whether inhibiting intestinal KLF5 weakens the beneficial role of RDN on gut microbiota. Sprague-Dawley rats were distributed into a CG (sham transverse aortic constriction [TAC] and sham RDN), HF (induced by TAC), or RDN (underwent RDN after TAC) group or a CG.M, HF.M, or RDN.M group, which included the administration of the KLF5 inhibitor to the CG, HF, or RDN group, respectively. Transmission electron microscopy, mRNA, and protein expression of KLF5 and desmoglein 2 (DSG2) in jejunum and sequencing of the 16S rRNA gene in fecal samples were evaluated. KLF5 expression was lower in the RDN group than in the HF group (*P *< 0.001). The microvillus length, density, length-to-width ratio, and DSG2 expression were lower in the RDN.M group than in the RDN group, and the same trend was observed between the HF.M and HF groups (all *P* < 0.05). The gut bacterial community structure was altered after administration of a KLF5 inhibitor. The abundances of *Proteobacteria*, *Gammaproteobacteria*, *Sutterella*, and *Prevotellaceae* were higher, and the abundance of *Firmicutes* was lower in the RDN.M group than in the RDN group (all *P* < 0.05). These findings indicated that RDN suppressed intestinal KLF5 expression, and inhibiting intestinal KLF5 expression exacerbated the gut microbiota by impairing the intestinal barrier function in HF rats following RDN, which weakened the beneficial role of RDN on gut microbiota.

**IMPORTANCE** Krüppel-like factor 5 (KLF5) is critical for the maintenance of intestinal barrier function. It is unclear whether intestinal KLF5 expression can be affected by renal denervation (RDN) in heart failure (HF) and whether inhibiting intestinal KLF5 expression exacerbates the gut microbiome and weakens the role of RDN in mitigating gut microbiome aberrations in HF rats after RDN. We demonstrated that RDN significantly suppressed intestinal KLF5 expression and that inhibiting intestinal expression of KLF5 exacerbated the gut microbiota and weakened the role of RDN in mitigating microbiota aberrations by impairing intestinal barrier function, resulting in an increase in bacteria harmful to cardiac function and a decrease in beneficial bacteria in HF rats following RDN. This study highlighted the important roles of intestinal KLF5 in modulating gut microbiota in HF and suggested that the influence of RDN on intestinal KLF5 was another possible role of RDN in HF besides downregulating the sympathetic nerve.

## INTRODUCTION

Heart failure (HF) is the end stage of various heart diseases. The prevalence of HF is escalating rapidly over time, with the aging of the population (1). HF causes considerable morbidity and mortality and has become a major contributor to reducing quality of life (2). While the pathogenesis of heart failure is not clearly defined, a growing body of evidence suggests that gut microbiome aberrations and intestinal dysfunction are potential contributors to the development of heart failure (3, 4). Gut mucosal permeability and gut dysbiosis are notably attributed to the pathogenesis of HF (5, 6).

Krüppel-like factor 5 (KLF5) is a zinc-finger transcription factor regulating a variety of biological processes (7). Cardiomyocyte KLF5 expression is increased in mice with myocardial infarction or patients with ischemic heart failure, and genetic or pharmacological inhibition of KLF5 can increase ejection fraction (8). Meanwhile, KLF5 is critical for intestinal development, homeostasis, and maintenance of intestinal barrier function (9, 10). It can also influence intestinal stem cells in both physiological and pathological conditions by regulating the epigenetic and transcriptional activities of intestinal stem cell-specific gene sets (11). However, KLF5 levels can be affected by many factors. Treatment of collecting duct cells isolated from mice with isoproterenol increased the expression of KLF5 (12), suggesting that KLF5 levels were related to sympathetic nerve activity. In addition, it has been reported that renal denervation (RDN), which downregulates sympathetic nerve activities by ablating renal sympathetic nerves, significantly suppresses renal induction of KLF5 in mice undergoing transverse aortic constriction (TAC) (12), showing that KLF5 levels may be influenced by RDN.

In our previous study, we demonstrated that RDN mitigates gut microbiota aberrations in rats with chronic heart failure (13). This beneficial role of RDN on gut microbiota is partially attributed to the fact that RDN improves intestinal barrier function and ameliorates intestinal dysbiosis (14). Although RDN suppressed renal KLF5 expression, it is unclear whether intestinal expression of KLF5 can also be affected by RDN in HF and whether inhibiting intestinal KLF5 expression exacerbates the gut microbiome and weakens the role of RDN in mitigating gut microbiome aberrations in HF rats after RDN. Therefore, the relationship between RDN, intestinal KLF5, and gut microbiota in HF was investigated in this study. The aim was to evaluate the influence of RDN on intestinal KLF5 expression and to assess the effects of inhibiting intestinal KLF5 on the gut microbiota and the affecting intestinal barrier function in HF rats after RDN.

## RESULTS

### RDN suppressed jejunal KLF5 expression in heart failure rats.

**FIG 1:**
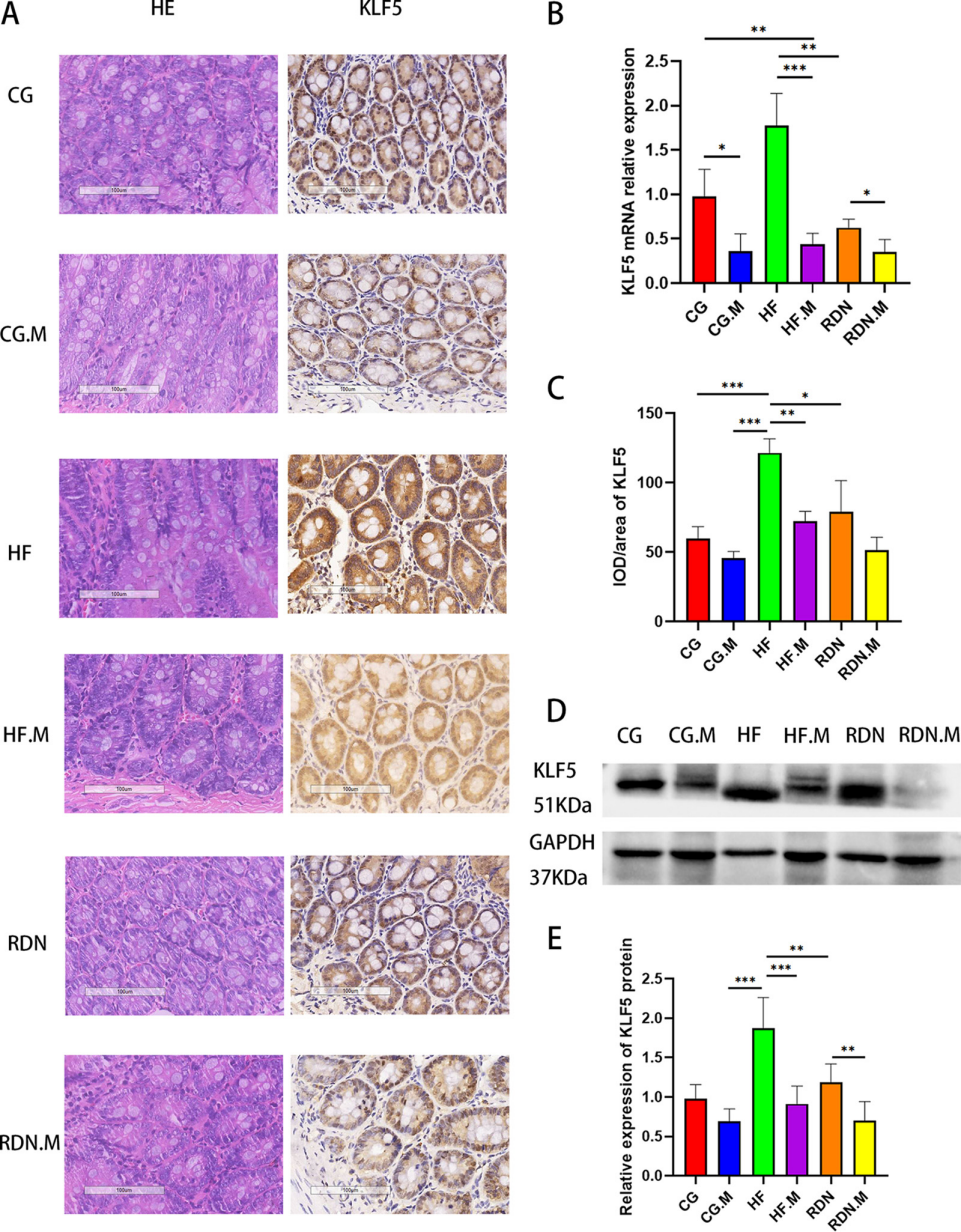
ML264 or RDN suppressed jejunal KLF5 expression in heart failure rats in distinct groups. (A) HE staining and immunohistochemistry of KLF5 (200×). (B) KLF5 mRNA relative expression. (C) Mean integrated optical density for KLF5. KLF5 protein expression detected by Western blotting (D) and the quantification of Western blotting (E). *, *P* < 0.05; **, *P* < 0.01; ***, *P* < 0.001.

Jejunal KLF5 protein expression was evaluated by immunohistochemistry and Western blot analysis. The integrated optical density (IOD)/area of the KLF5 protein in the HF.M group (72.45 ± 7.02 versus 121.52 ± 10.19; *P = *0.002) and RDN group (79.34 ± 22.17 versus 121.52 ± 10.19; *P = *0.040) was lower than that in the HF group (Fig. 1A and C). The relative mRNA expression of jejunal KLF5 was lower in the CG.M group than in the CG group (0.36 ± 0.19 versus 0.98 ± 0.30; *P = *0.029), in the HF.M group than in the HF group (0.44 ± 0.12 versus 1.78 ± 0.36; *P* < 0.001), and in the RDN.M group than in the RDN group (0.35 ± 0.14 versus 0.63 ± 0.09; *P = *0.049). Furthermore, KLF5 mRNA levels decreased significantly in the RDN group compared to those in the HF group (0.63 ± 0.09 versus 1.78 ± 0.36; *P = *0.003) (Fig. 1B).

Western blot analysis also showed that the relative expression of KLF5 protein in the HF.M group (0.91 ± 0.23 versus 1.87 ± 0.39; *P* < 0.001) and RDN group (1.18 ± 0.24 versus 1.87 ± 0.39; *P = *0.004) was decreased compared with that in the HF group. The RDN.M group possessed lower relative expression of KLF5 protein than the RDN group (0.70 ± 0.24 versus 1.18 ± 0.24; *P = *0.006) (Fig. 1D and E).

### Inhibition of KLF5 damaged the gut mucosa function of heart failure rats.

**FIG 2:**
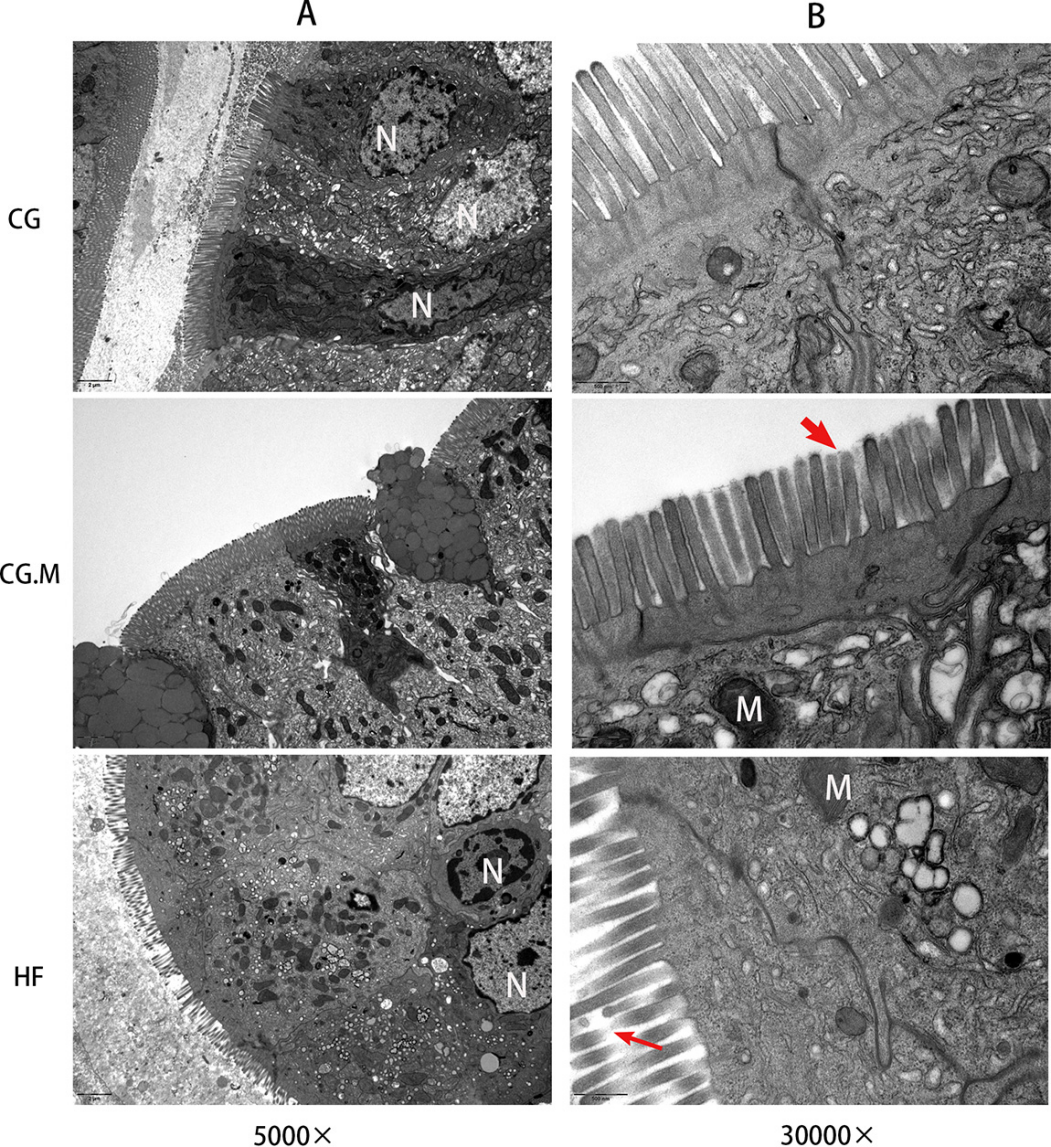
Pharmacological inhibition of KLF5 led to intestinal mucosal dysfunction in normal or heart failure rats. Transmission electron microscopy images showed that microvilli were either damaged (thin arrow) or became shorter (thick arrow), and the tight junctions of intestinal epithelial cells were disrupted (5,000× [A]; 30,000× [B]). N, nucleus; M, mitochondria.

**FIG 3:**
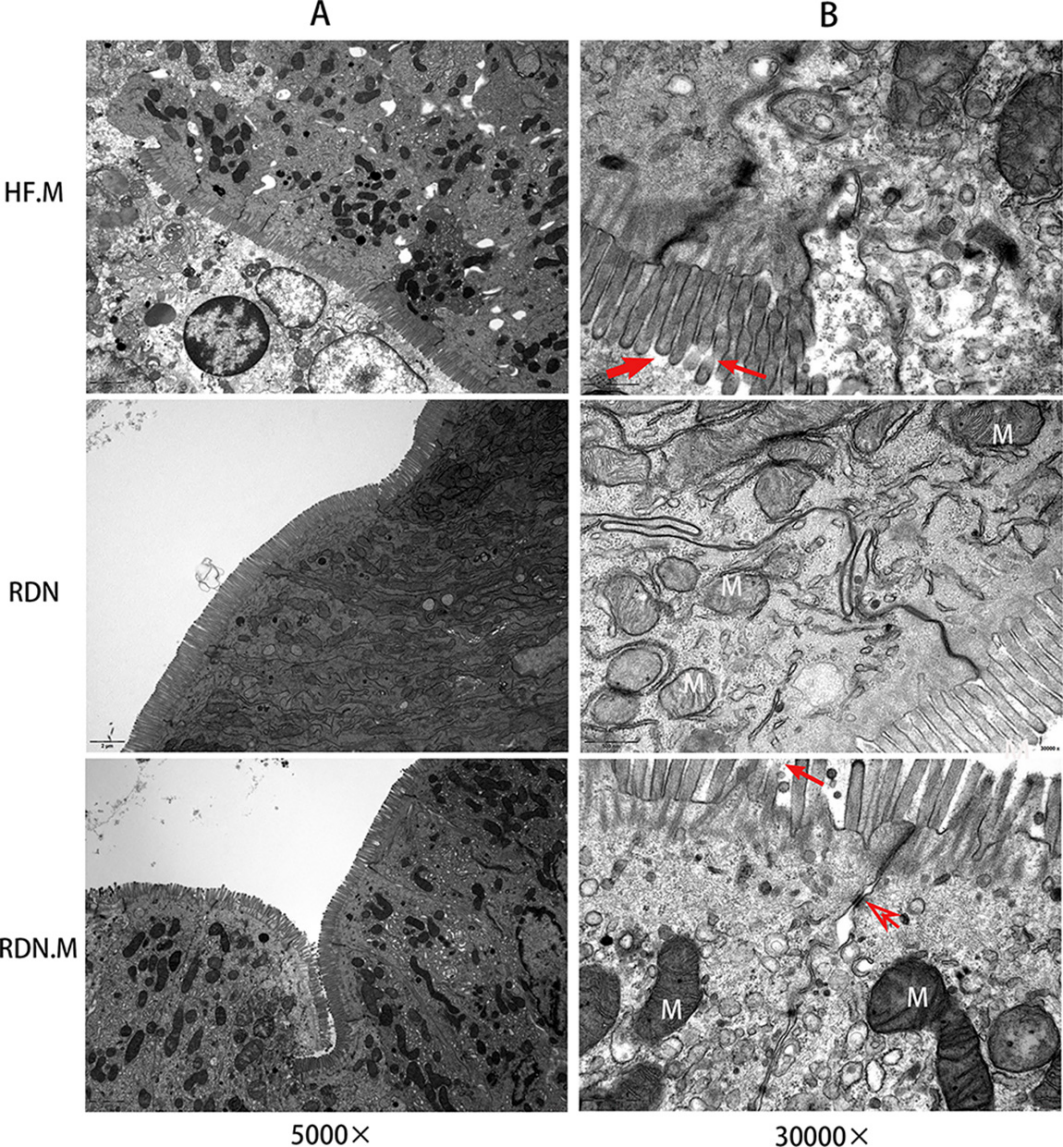
Pharmacological inhibition of KLF5 led to intestinal mucosa dysfunction in heart failure rats after or without RDN. Transmission electron microscopy images showed that microvilli were either damaged (thin arrow) or became shorter (thick arrow), and the tight junctions of intestinal epithelial cells were disrupted (open arrow) (5,000× [A]; 30,000× [B]). N, nucleus; M, mitochondria.

**FIG 4:**
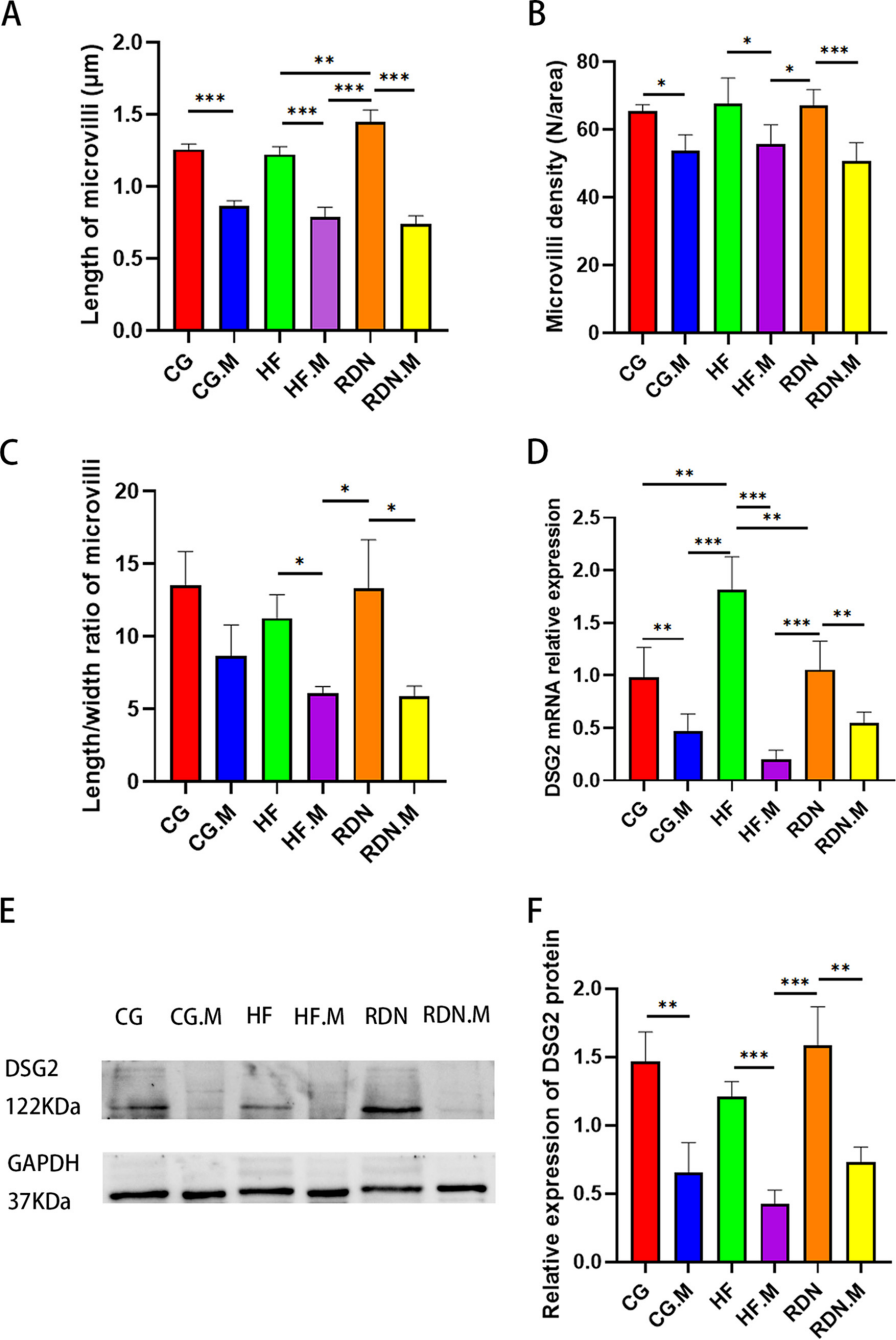
Pharmacological inhibition of KLF5 led to intestinal mucosa dysfunction in heart failure rats. (A) Length of microvilli in separate groups. (B) Density of microvilli in separate groups. (C) The ratio of microvillus length to width in separate groups. (D) Relative DSG2 mRNA expression. (E) DSG2 protein expression detected by Western blotting and (F) the quantification of Western blotting. *, *P* < 0.05; **, *P* < 0.01; ***, *P* < 0.001. DSG2, desmoglein 2.

Transmission electron microscopy (TEM) was used to observe microvilli, intestinal epithelial cells, and their connections. The microvillus structure was significantly changed. After the administration of ML264, microvilli were either damaged or became shorter, and the tight junctions of intestinal epithelial cells were disrupted (Fig. 2 and 3). The microvillus length in the CG.M group was significantly smaller than that of the CG group (0.87 ± 0.03 versus 1.26 ± 0.04; *P* < 0.001). It was also significantly lower in the HF.M group than in the HF group (0.79 ± 0.06 versus 1.22 ± 0.05; *P* < 0.001) and in the RDN.M group than in the RDN group (0.74 ± 0.06 versus 1.45 ± 0.08; *P* < 0.001) (Fig. 4A). In addition, the density of microvilli was significantly smaller in the CG.M group than in the CG group (53.70 ± 4.75 versus 65.40 ± 1.96; *P = *0.003), in the HF.M group than in the HF group (55.70 ± 5.7 versus 67.70 ± 7.45; *P = *0.021), and in the RDN.M group than in the RDN group (50.72 ± 5.39 versus 67.15 ± 4.65; *P* < 0.001) (Fig. 4B). The same trend was also observed in the ratio of microvillus length to width (Fig. 4C).

Desmoglein 2 (DSG2) is a major component of the gene encoding desmosome structures, which is important in maintaining intestinal barrier function. DSG2 relative mRNA expression in jejunal tissue was lower in the CG.M group than in the CG group (0.47 ± 0.16 versus 0.98 ± 0.29; *P = *0.003), in the HF.M group than in the HF group (0.20 ± 0.09 versus 1.81 ± 0.32; *P* < 0.001), and in the RDN.M group than in the RDN group (0.55 ± 0.10 versus 1.06 ± 0.27; *P = *0.002) (Fig. 4D). Similarly, Western blot analysis also showed that jejunal DSG2 protein expression normalized to GAPDH (glyceraldehyde-3-phosphate dehydrogenase) in rats was also reduced after administration of a KLF5 inhibitor (CG.M versus CG [0.65 ± 0.22 versus 1.46 ± 0.22], *P = *0.002; HF.M versus HF [0.42 ± 0.10 versus 1.21 ± 0.11], *P* < 0.001; RDN.M versus RDN [0.73 ± 0.11 versus 1.59 ± 0.28], *P* < 0.001) (Fig. 4E and F).

### The microbial community structure varied among groups.

**FIG 5:**
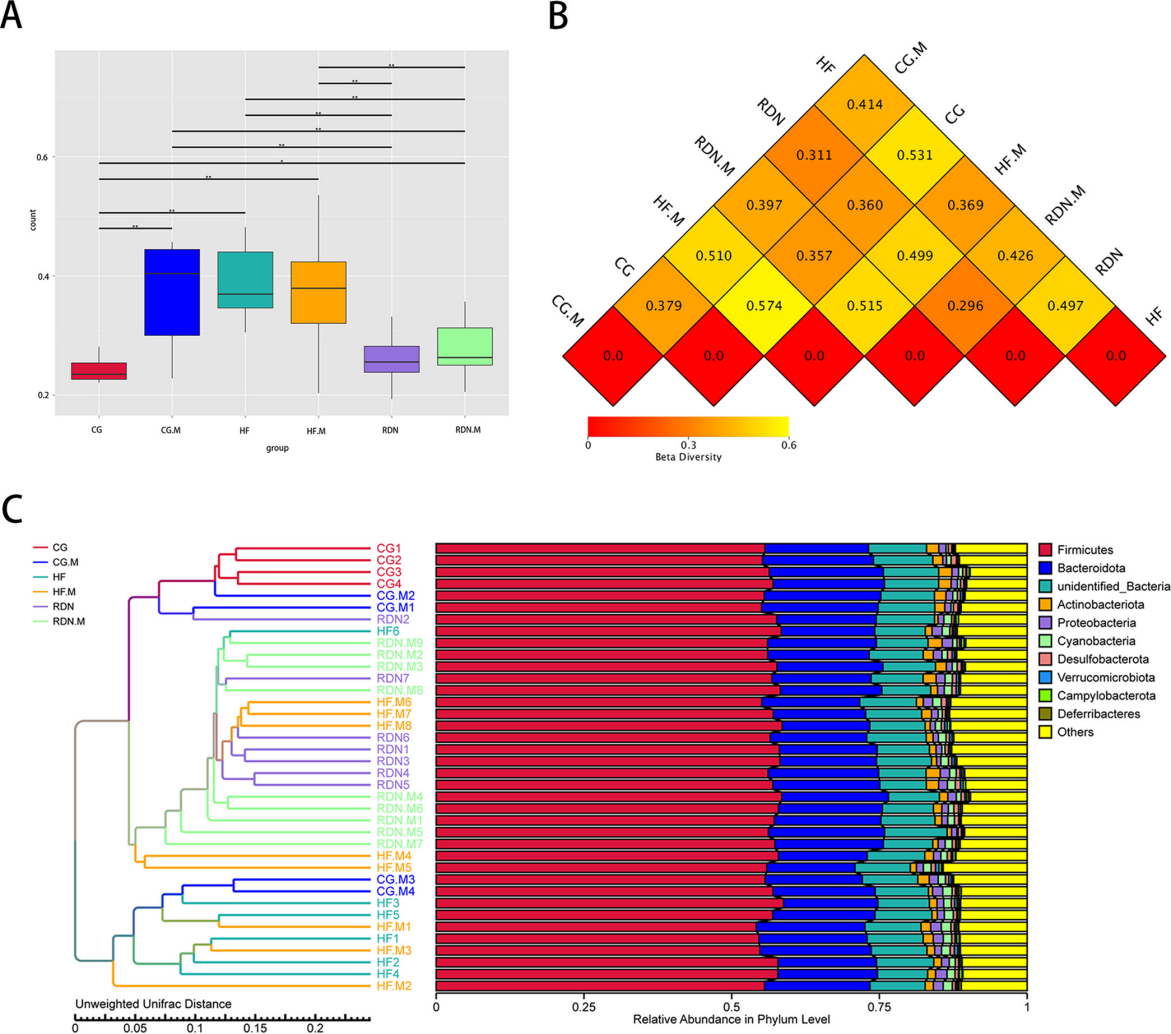
Microbial community structure varied among the distinct groups. (A) Beta diversity based on unweighted UniFrac distance. (B) Dissimilarity coefficient of the beta diversity heatmap based on the unweighted UniFrac distance of groups. (C) UPGMA clustering tree based on the unweighted UniFrac distance. *, *P* < 0.05; **, *P* < 0.01.

Fig. 5A shows a comparison of beta diversity, which refers to the variance among groups, based on unweighted UniFrac distance among the CG, CG.M, HF, HF.M, RDN, and RDN.M groups. There was a significant difference in the beta diversity community composition between the CG and CG.M groups, CG and HF groups, and HF and RDN groups (all *P* < 0.05). These data revealed that the bacterial communities varied among groups.

The numbers in the grids of Fig. 5B are the dissimilarity coefficients based on unweighted UniFrac distances among groups. The difference in microbial community structure between the two groups was proportional to the dissimilarity coefficient. The dissimilarity coefficient between the HF and CG groups was 0.531, while it was 0.497 between the HF and RDN groups. In addition, the dissimilarity coefficient between the RDN and RDN.M groups was 0.296 and between the HF and HF.M groups was 0.369. These results indicated that the gut microbial community structure was changed in HF rats compared with normal rats and could also be altered by ML264 or RDN treatment.

The unweighted pair group method with arithmetic mean (UPGMA) clustering tree based on unweighted UniFrac distance exhibited similarity between samples (Fig. 5C). In the clustering tree, samples with similar community structures tended to cluster together; the closer two samples were located, the more similar their microbiota community compositions were. The HF group displayed the greatest distance from the CG group, which indicated the largest community difference. The relatively small distance between the CG, RDN.M, HF.M, and RDN groups indicated a similar community structure. Analysis of molecular variance (AMOVA) based on weighted UniFrac distance was also employed to identify the differences in bacterial community structure among groups. ANOVA showed that there were statistically significant differences between the CG and HF groups (*P = *0.019), the HF and RDN groups (*P = *0.01), the CG and CG.M groups (*P = *0.04), and the HF and HF.M groups (*P = *0.04). The results revealed that the bacterial community structure changed in rats suffering from heart failure, RDN, or ML264 administration.

### Composition of gut microbiota at the phylum level.

**FIG 6:**
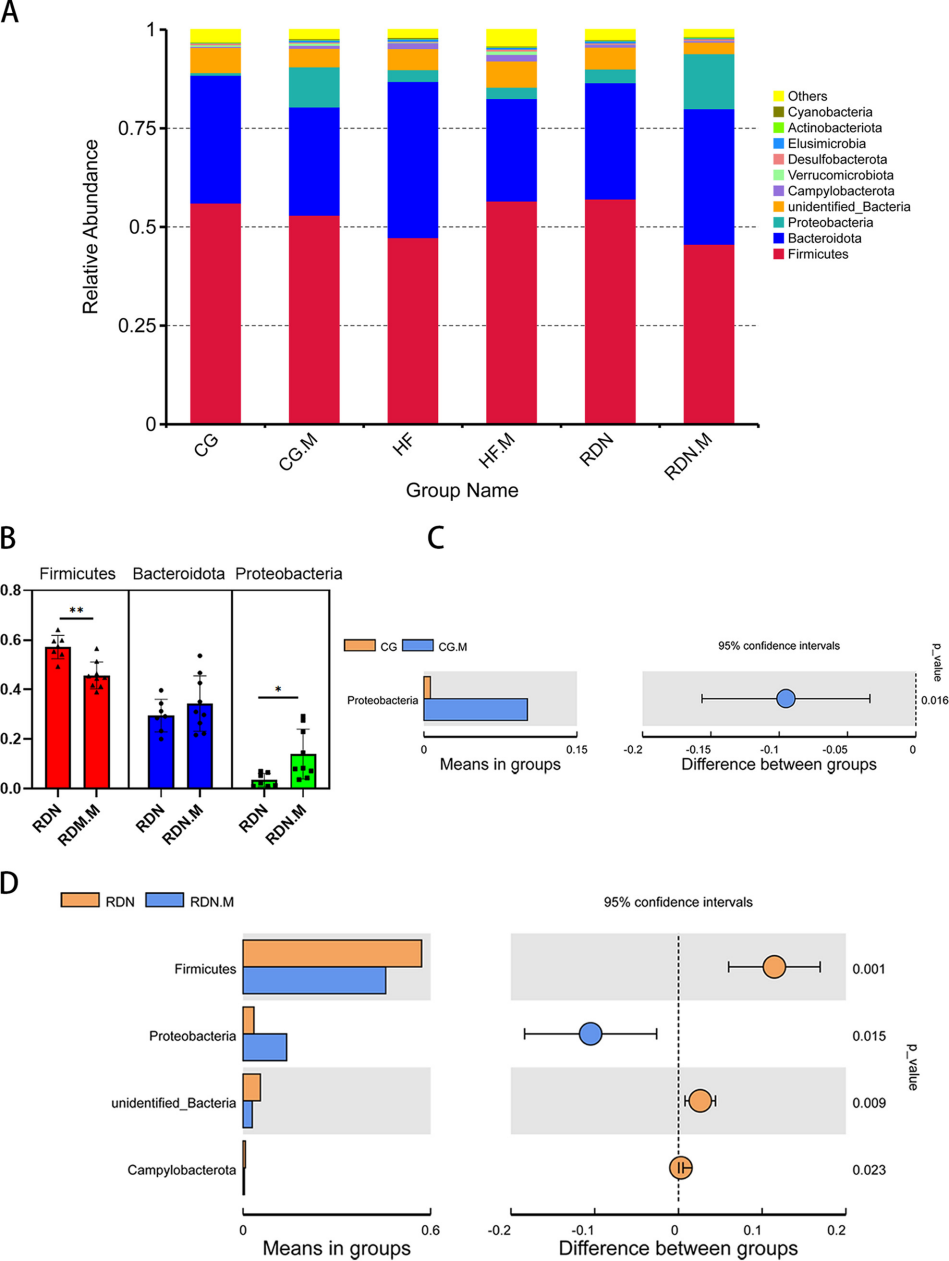
Composition of gut microbiota at the phylum level. (A) Relative abundance distribution of the top 10 phyla in the CG and CG.M, HF, HF.M, RDN, and RDN.M groups at the phylum level. (B) Comparison of the relative abundance of the phyla *Firmicutes*, *Bacteroidota*, and *Proteobacteria* between RDN and RDN.M groups. (C) Distinct bacterial phyla in the CG group (yellow) and the CG.M group (blue). (D) Distinct bacterial phyla in the RDN group (yellow) and the RDN.M group (blue). *, *P* < 0.05; **, *P* < 0.01.

At the phylum level, *Firmicutes* and *Bacteroidota* were the main predominant phyla present in all groups. The abundance of *Bacteroidota* was increased in the HF group (39.59%) compared with that in the CG group (32.39%), and it was decreased in the RDN (29.46%) and RDN.M (34.34%) groups. Meanwhile, the abundance of the phylum *Firmicutes* showed the opposite trend to the phylum *Bacteroidota* (Fig. 6A).

The abundance of *Proteobacteria* in the CG.M group was higher than in the CG group (*P = *0.016) and was higher in the RDN.M group than in the RDN group (*P = *0.015) (Fig. 6B and C). However, the abundance of *Firmicutes* in the RDN group was significantly higher than that in the RDN.M group (*P = *0.001) (Fig. 6B and D). The *Firmicutes*/*Bacteroidota* (F/B) ratio was increased in RDN (1.94) and RDN.M (1.33) groups when compared with that in the HF group (1.20).

### Composition of gut microbiota at the class level.

**FIG 7:**
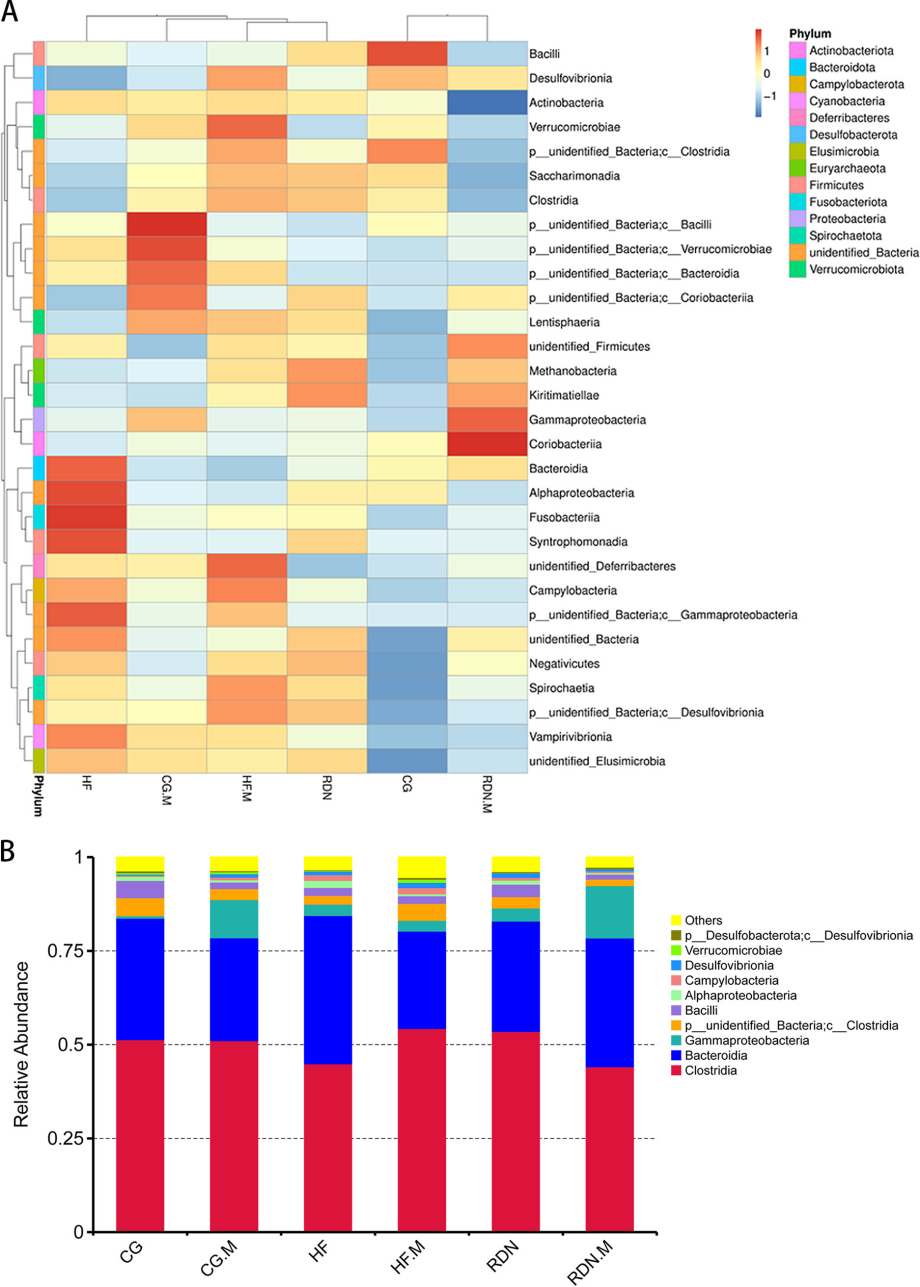

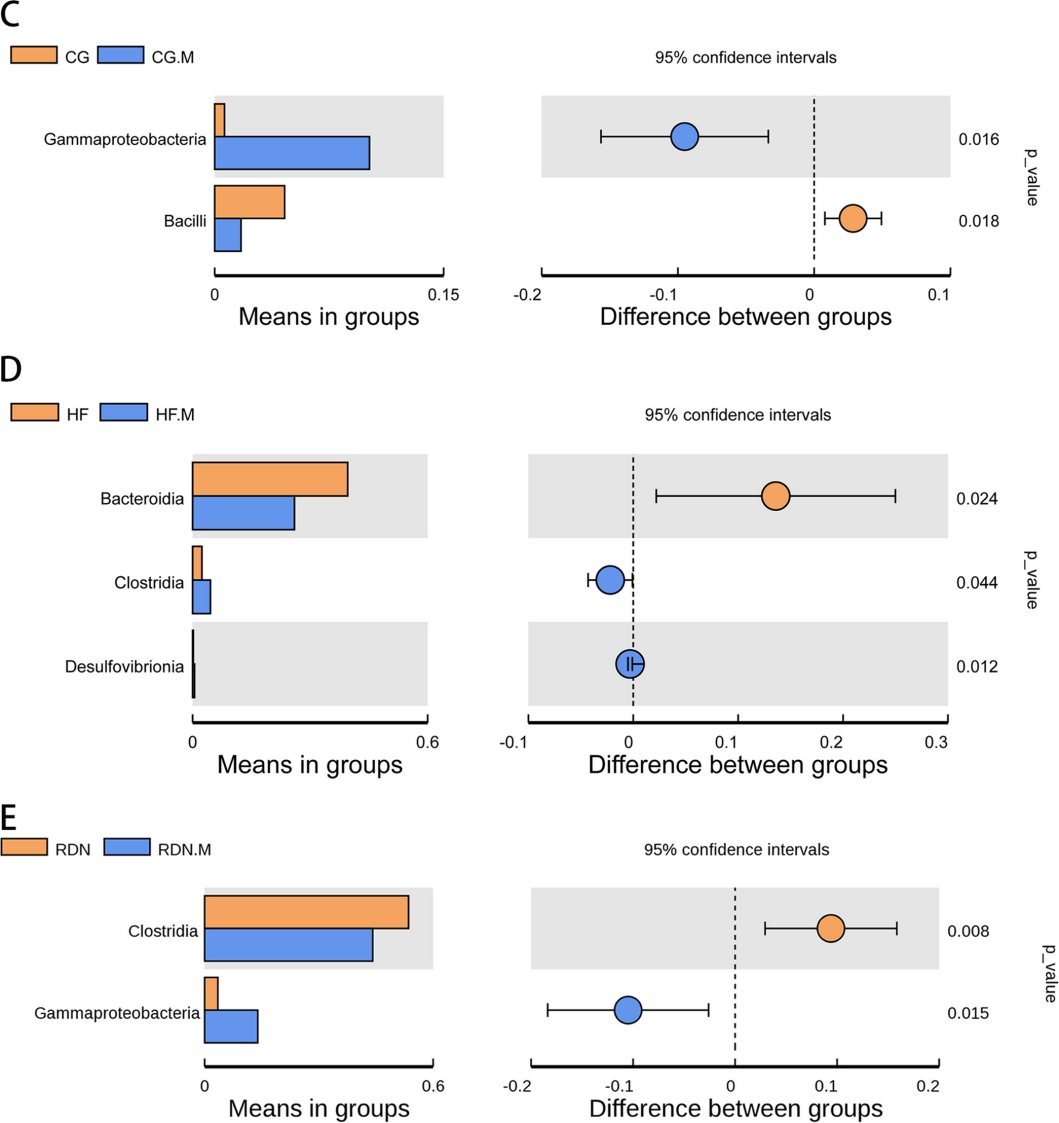
Composition of gut microbiota at the class level. (A) Heatmap of the top 35 bacteria in the C.G., C.G.M., H.F., H.F.M., R.D.N., and R.D.N.M. groups at the class level. (B) Relative abundance distribution of the top 10 bacteria in the C.G., C.G.M., H.F., H.F.M., R.D.N., and R.D.N.M. groups at the class level. (C) Distinct bacterial classes in the C.G. group (yellow) and the C.G.M. group (blue). (D) Distinct bacterial classes in the H.F. group (yellow) and the H.F.M. group (blue). (E) Distinct bacterial classes in the RDN group (yellow) and the R.D.N.M. group (blue). *P* < 0.05 as per *t* test.

As shown in the heatmap of the top 35 bacteria in the CG, CG.M, HF, HF.M, RDN, and RDN.M groups (Fig. 7A), the abundance of *Bacteroidia* was increased in the HF group and decreased in the HF.M group, while the abundance of *Gammaproteobacteria* was increased in the RDN.M group and decreased in the RDN group.

As illustrated in Fig. 7B, the HF group showed more *Bacteroidia* than the CG group, whereas the HF.M group had more *Clostridia* and the RDN group had fewer *Bacteroidia* than the HF group. In addition, *Gammaproteobacteria* in the CG.M group were more abundant than in the CG group (*P = *0.016) and higher in the RDN.M group than in the RDN group (*P = *0.015) (Fig. 7C and E)*. Clostridia* were higher in the HF.M group than in the HF group (*P = *0.044) (Fig. 7D).

### Composition of gut microbiota at the genus level.

**FIG 8:**
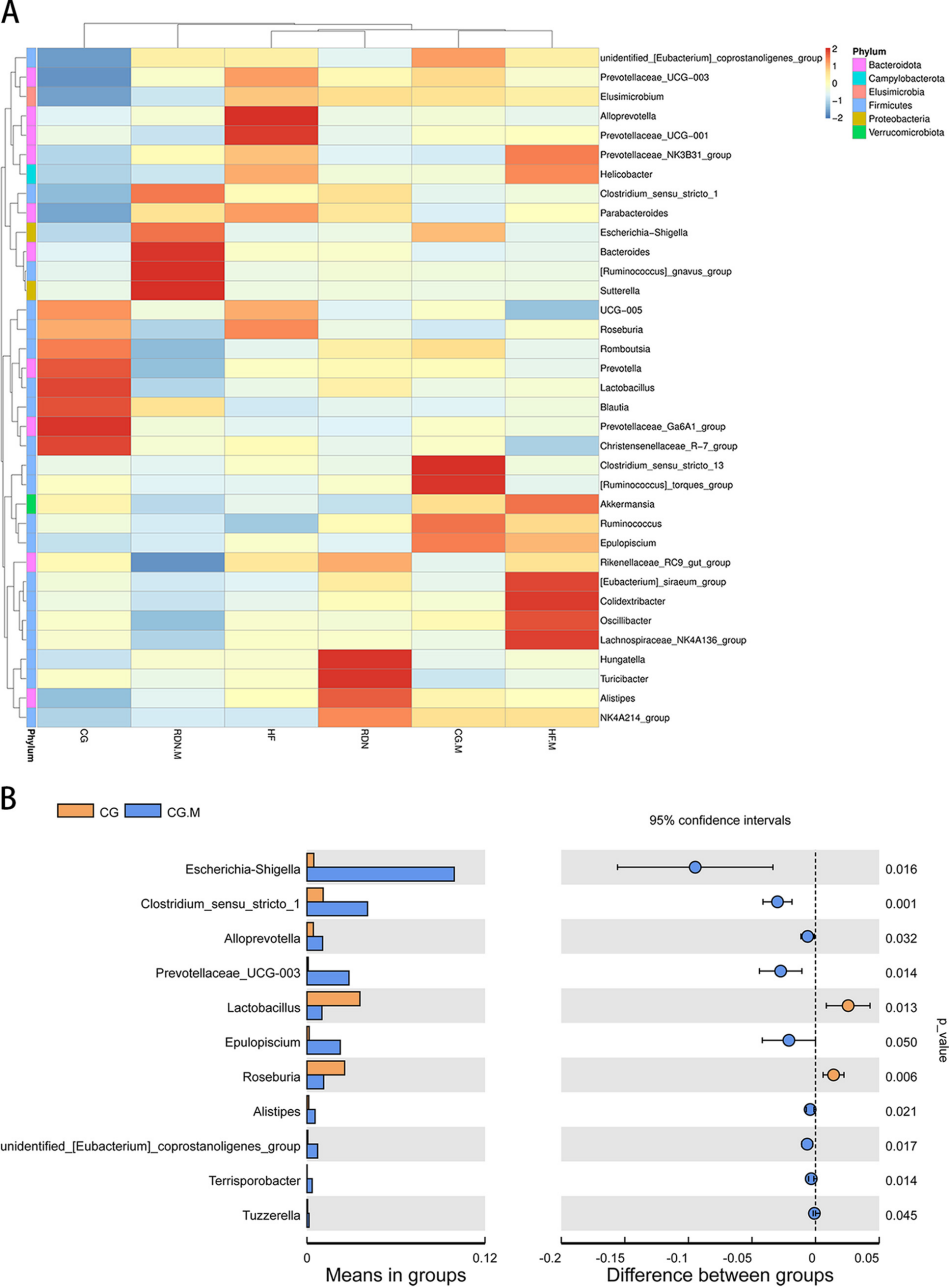

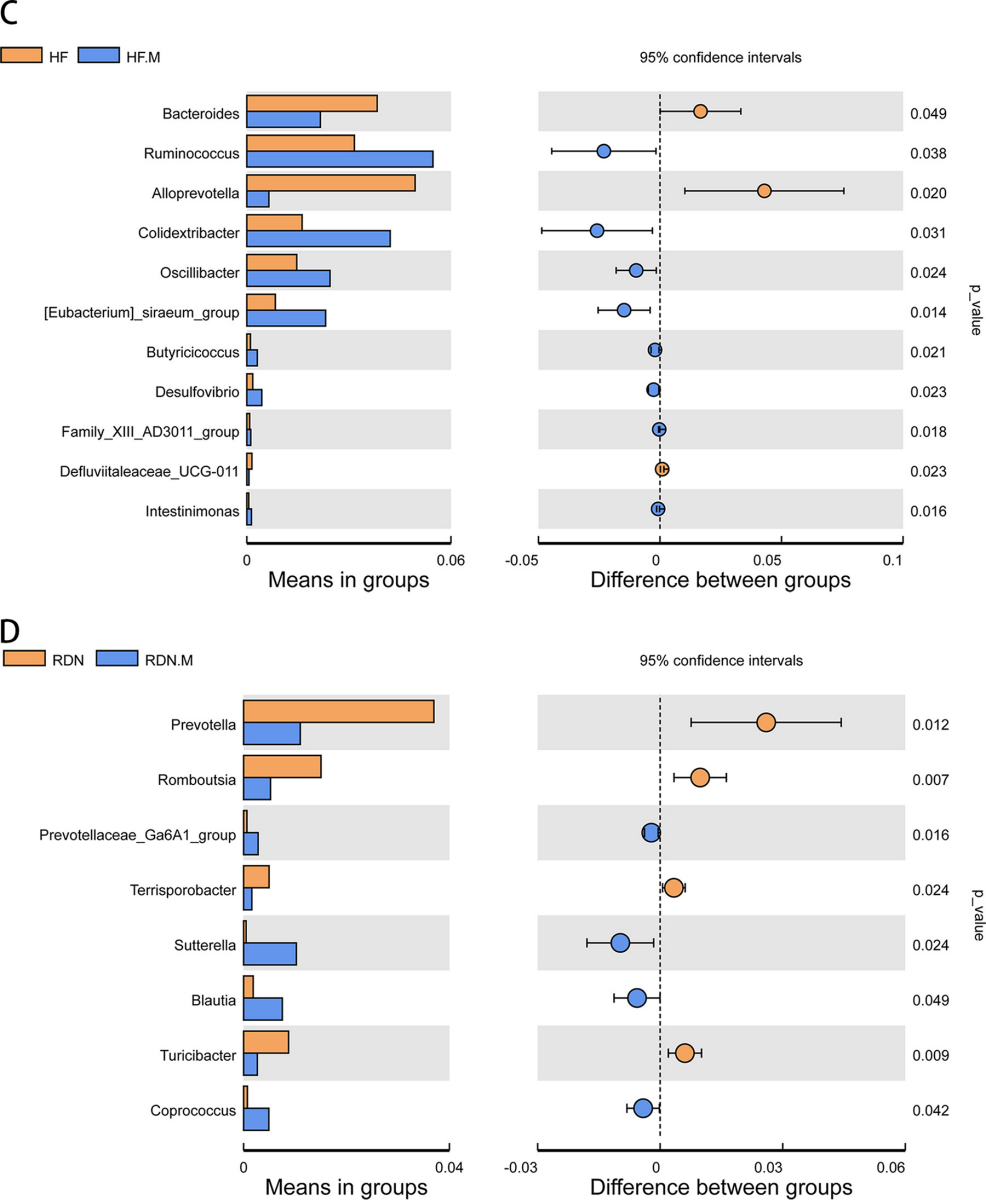
Composition of gut microbiota at the genus level. (A) Heatmap of the top 35 bacteria in the C.G., C.G.M., H.F., H.F.M., R.D.N., and R.D.N.M. groups at the genus level. (B) Distinct bacterial genera in the CG group (yellow) and the C.G.M. group (blue). (C) Distinct bacterial genera in the H.F. group (yellow) and the H.F.M. group (blue). (D) Distinct bacterial genera in the RDN group (yellow) and the R.D.N.M. group (blue). *P* < 0.05 as per *t* test.

By comparison with the top 35 most abundant genera, rats in the CG group exhibited 6 abundant genera (red color) within *Firmicutes* and 2 abundant genera within *Bacteroidota*, whereas rats in the HF group possessed 5 abundant genera within *Bacteroidota* and 2 abundant genera within *Firmicutes*. It was obvious that the HF group showed an increased abundance of *Alloprevotella* and *Prevotellaceae* UCG-001, whereas the RDM.M group exhibited overgrowth of *Bacteroides* and *Sutterella* (Fig. 8A).

However, the abundance of *Clostridium* and *Prevotellaceae* UCG-003 in the CG.M group was higher than that in the CG group (*P = *0.001 and 0.014) (Fig. 8B). The abundance of *Ruminococcus* in the HF.M group was higher than that in the HF group (*P = *0.038) (Fig. 8C). Furthermore, RDN.M group had more *Sutterella* (*P = *0.024) and *Prevotellaceae* (*P = *0.016) than the RDN group (Fig. 8D).

**FIG 9:**
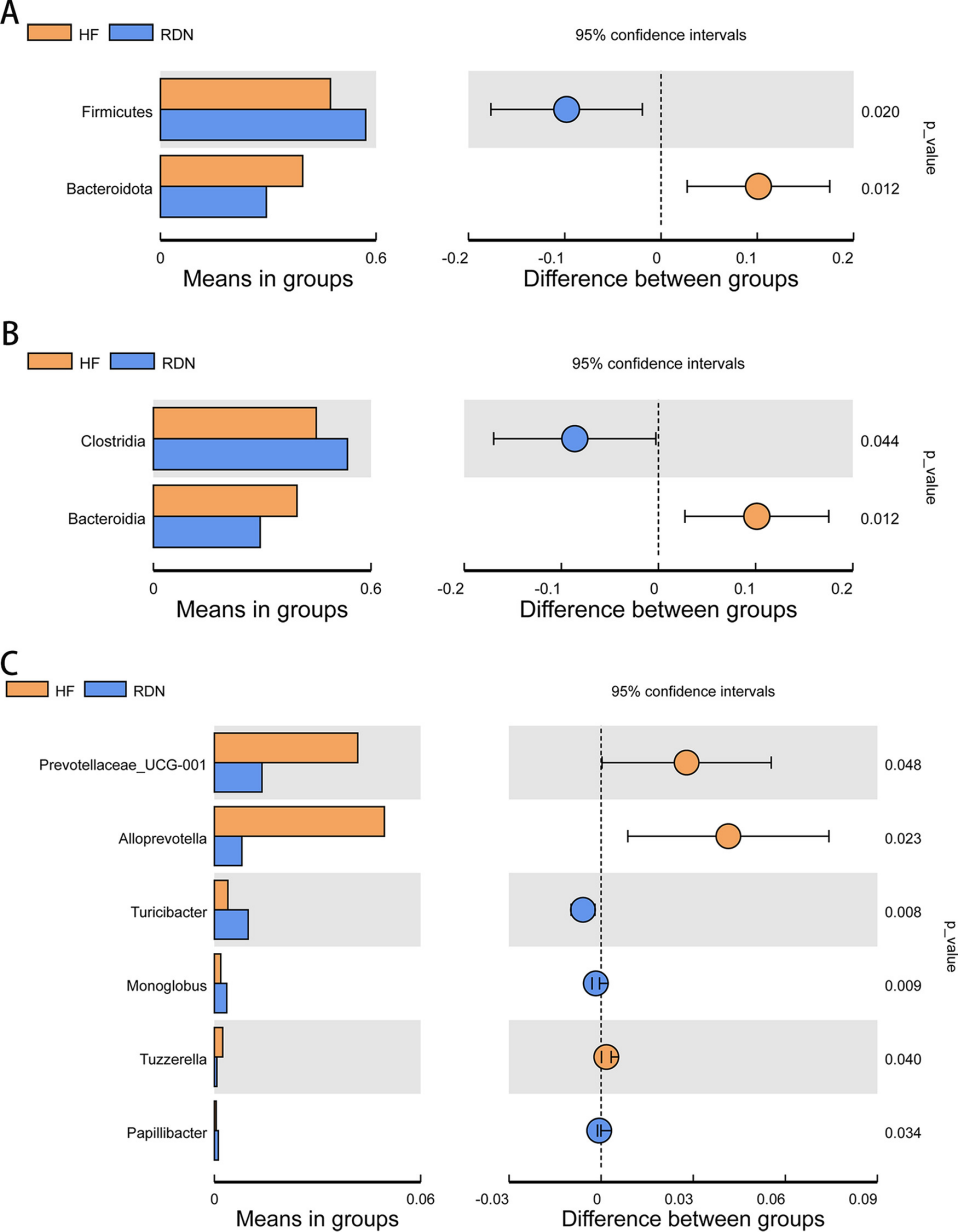
Composition of gut microbiota between the HF and RDN groups at various levels. (A) Distinct bacterial phyla in the HF group (yellow) and the RDN group (blue). (B) Distinct bacterial classes in the HF group (yellow) and the RDN group (blue). (C) Distinct bacterial genera in the HF group (yellow) and the RDN group (blue).

In addition, intestinal microbiota changes were also observed between the HF and RDN groups to determine the effect of the decrease in intestinal KLF5 levels after RDN on intestinal microbiota. The results of the *t* test showed that the abundance of *Bacteroidota* was significantly higher and *Firmicutes* was significantly lower in the HF group than in the RDN group (*P = *0.012 and 0.020) at the phylum level (Fig. 9A). The RDN group had lower *Bacteroidia* and *Prevotellaceae* UCG-001 than the HF group at the class (*P = *0.012) (Fig. 9B) and genus levels, respectively (Fig. 9C) (*P = *0.048).

### Predictive function of microbial communities.

**FIG 10:**
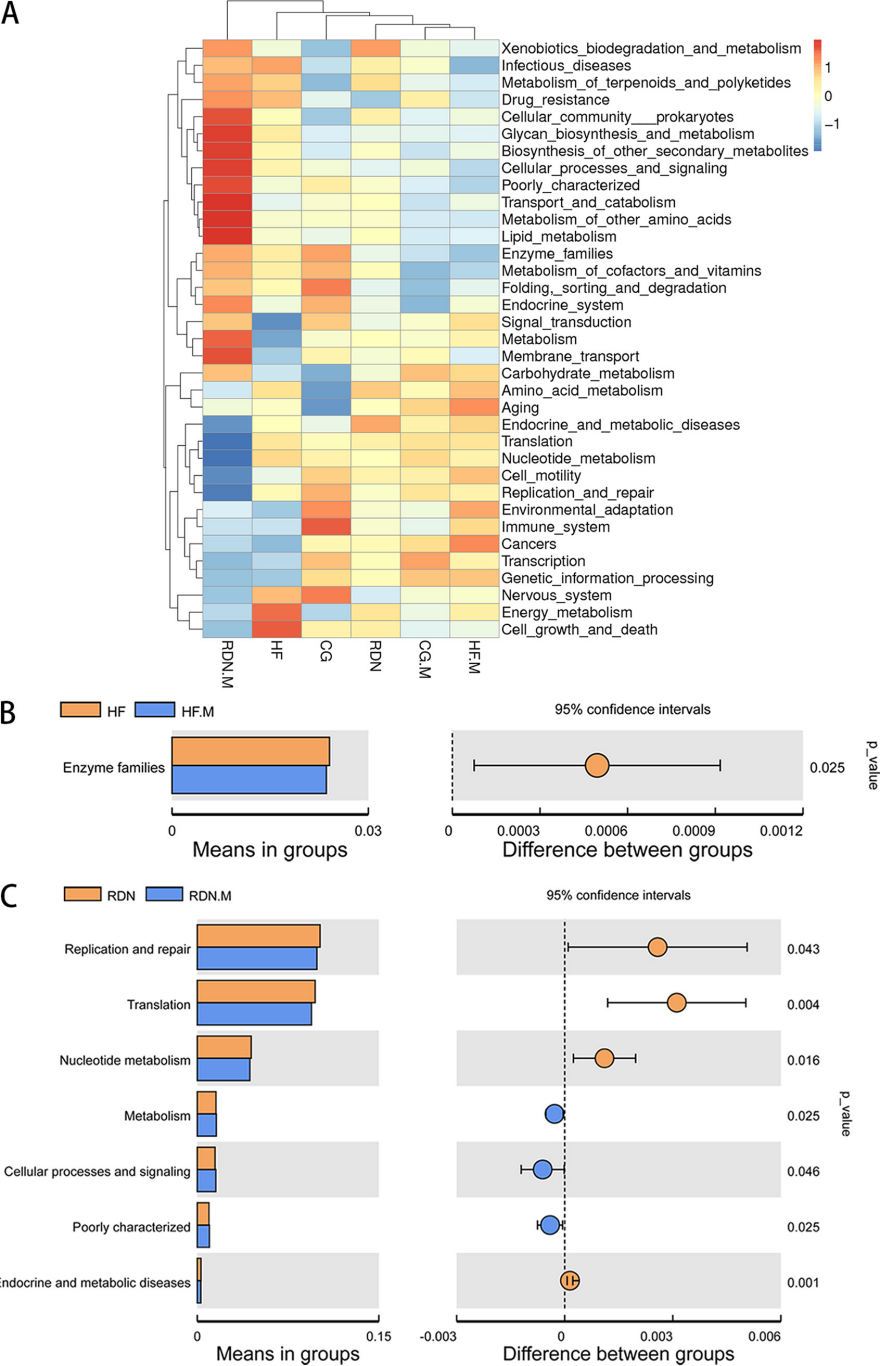
Predictive functional profiling of microbial communities by Tax4Fun analysis. (A) A heatmap was made based on Tax4Fun functional annotations. (B) Comparison of enriched metabolic pathways between the HF and HF.M groups. (C) Comparison of enriched metabolic pathways between RDN and RDN.M groups. *P* < 0.05 as per *t* test.

Predictive functional profiling of microbial sequencing data was annotated based on the KEGG database using Tax4Fun analysis. As shown in Fig. 10A, the abundances of functional categories based on KEGG (level 2) between separate groups were analyzed. The abundances of translation and nucleotide metabolism were significantly decreased in the RDN.M group compared with those in the other groups. The abundance of pathways associated with enzyme families was significantly lower in the HF.M group than in the HF group (*P = *0.025) (Fig. 10B). Furthermore, replication and repair were significantly lower (*P = *0.043), and metabolism (*P = *0.025) and cellular processes and signaling (*P = *0.046) were significantly higher in RDN.M group than in the RDN group (Fig. 10C).

## DISCUSSION

Intestinal KLF5 is associated with intestinal barrier function, which is critically involved in the development and maintenance of heart failure. In the present study, the RDN group had significantly lower expression of jejunal KLF5 than the HF group. The microvillus length, density, and ratio of length to width were significantly decreased, and the protein and mRNA expression of DSG2 were lower in the HF.M group than in the HF group. The same trend was also observed between the RDN.M and RDN groups. Gut bacterial community structure and the abundance of *Firmicutes*, *Proteobacteria*, *Gammaproteobacteria*, *Sutterella*, *Clostridia*, and *Prevotellaceae* UCG were altered after administration of a KLF5 inhibitor in rats with HF. Predictive functional profiling of microbial communities showed that replication and repair, translation, and nucleotide metabolism were significantly lower, and metabolism and cellular processes and signaling were significantly higher in the RDN.M group than in the RDN group. These findings indicated that RDN significantly suppressed intestinal expression of KLF5 in HF rats induced by TAC, and inhibiting intestinal expression of KLF5 impaired the intestinal barrier function, resulting in an increase in intestinal bacteria harmful to cardiovascular health and a decrease in beneficial bacteria.

Krüppel-like factor family (KLF) members have the ability to regulate intrinsic axon growth (15). KLF5 is an important neural growth-suppressive KLF (16), and its levels may be regulated by nervous stimulants. It was reported that treatment of collecting duct cells with isoproterenol increased the expression of KLF5 (12), suggesting that KLF5 levels were related to sympathetic nerve activity. It is possible that increased sympathetic nerve activity reflexively enhanced the expression of KLF5 because KLF5 could inhibit neural growth, which avoided further increase in sympathetic nerve activity. Therefore, RDN, which reduces renal and systemic sympathetic nerve activity by the ablation of renal nerves (17), significantly suppressed renal induction of KLF5 in mice undergoing TAC (12). Our results showed that jejunal KLF5 levels could also be suppressed by RDN. In addition to the kidney, KLF5 levels in other organs, especially in the state of sympathetic nerve overactivity, may also be regulated by RDN because of a pathophysiological link between the kidney and other organs through sympathetic nerves.

KLF5 is critical for the maintenance of intestinal barrier function. In this study, the microvillus length, density, and ratio of length to width were significantly decreased after administration of a specific KLF5 inhibitor, not only between the HF.M and HF groups but also between the RDN.M and RDN groups. DSG2 levels were also reduced after treatment with a KLF5 inhibitor, suggesting that microvillus integrity and function were impaired with the decrease in KLF5. These results indicated that KLF5 was a crucial factor in the maintenance of intestinal barrier function in HF, and a decrease in KLF5 led to damage to intestinal barrier function. Structural changes in the intestinal barrier result in functional alterations, including decreased intestinal absorption function and increased mucosal permeability (5, 18). This allows gut microbes and their products harmful to cardiac function to enter circulation more easily, resulting in deterioration of HF. Trimethylamine (TMA) is one of these products, which is the precursor of trimethylamine oxide (TMAO), and both can exert a negative impact on cardiomyocyte viability and cardiac function (19, 20).

Regarding the microbial community composition, our results showed that the abundance of the phylum *Firmicutes*, which had butyrate as their primary metabolic end product and exerted local anti-inflammatory effects in the gut wall (21), in the RDN.M group was lower than that in the RDN group. The F/B ratio was decreased in the RDN.M group (1.33) compared to that in the RDN group (1.94). The decrease in *Firmicutes* and the imbalance of the F/B ratio could lead to abnormal energy supply (22–24), which may contribute to the pathogenesis and progression of heart failure (25).

Additionally, the abundances of *Proteobacteria*, which have been shown to be related to an increased risk of cardiovascular disease (26) and also to be associated with TMA production (27), and *Gammaproteobacteria*, which also have a relationship with TMA production (28), were increased in HF rats administered a KLF5 inhibitor. Moreover, the RDN.M group had more *Sutterella* and *Prevotellaceae* than the RDN group. *Sutterella* was increased in patients with coronary heart disease and associated with obesity (29) and diabetes (30), and it could also impair the function of the intestinal antibacterial immune response (31) by regulating the levels of interleukin-13 transcripts (32), which is an important relevant cytokine that is known to affect epithelial barrier integrity (31). *Prevotellaceae* are also considered to be harmful to the cardiovascular system and have been reported to be significantly associated with cardiovascular disease risk (33). Further, the *Prevotellaceae* were reported to be the most important bacterial family associated with body mass index and obesity (34) and to be related to an elevated level of circulating succinate concomitant with impaired glucose metabolism in obese people (35).

In addition, the HF.M group had elevated levels of *Clostridia* and *Ruminococcus* than the HF group. *Clostridia* play a vital role in the maintenance of gut homeostasis (36). It was reported that the abundance of *Clostridia* was higher in patients with coronary heart disease (37, 38) and atherosclerotic cardiovascular disease (ACVD) (39). Moreover, ACVD-enriched bacteria, including *Clostridium*, positively correlated with diastolic or systolic blood pressure (39). *Ruminococcus* was also associated with higher TMAO levels (40). Furthermore, *Gammaproteobacteria*, *Prevotellaceae*, and *Clostridia* were also increased even in non-HF rats after administration of a KLF5 inhibitor. These results indicated that the decrease in intestinal KLF5 caused an increase in harmful intestinal bacteria and a decrease in gut bacteria beneficial to cardiac function. In this view, cardiac function may be affected by intestinal KLF5 through influencing gut microbiota.

In addition to microbial community composition, predictive functional analysis showed that specific metabolic pathways could be affected by KLF5 inhibitors. It was reported that short-chain fatty acids (SCFA) produced by gut microflora showed obvious benefits to the prognosis of heart failure and could reduce inflammation (41–43). This may help to partly explain the mechanism of the influence of intestinal KLF5 on heart failure. Future studies, especially on SCFA pathways, are warranted to validate this finding.

However, it remains unclear how RDN could still mitigate gut microbiota aberrations and improve cardiac function in rats with heart failure considering the role of RDN in decreasing intestinal KLF5. Although the decrease in intestinal KLF5 was harmful to intestinal barrier function and microbiota, which may impair cardiac function, the beneficial role of RDN on HF may be far more significant than the disadvantage of decreased intestinal KLF5 expression caused by RDN. In this view, enabling increasing and/or avoiding decreasing intestinal KLF5 expression is a potential target for enhancing the efficacy of RDN in HF.

Several limitations should be discussed. First, we investigated only the role of inhibiting intestinal KLF5 expression on intestinal barrier function and gut microbiota in HF. The influence of increasing intestinal KLF5 expression was not assessed. Whether increasing intestinal KLF5 expression is beneficial to intestinal barrier function and gut microbiota, contributing to improving HF and the therapeutic role of RDN in HF, remains unclear. Second, the effect of KLF5 on interepithelial lymphocytes (IELs) in the gut was not investigated in this study. It has been reported that the function of IELs is also vital to the maintenance of the intestinal barrier and physiological inflammation (44). Finally, some critical bacterial products related to HF, such as TMAO, were not detected. Perhaps the bacterial products can also be influenced by intestinal KLF5 because the microbial products may be more important to HF than the bacteria themselves.

In conclusion, our results showed that RDN suppressed intestinal expression of KLF5 in HF rats and that inhibiting intestinal expression of KLF5 exacerbated the gut microbial community structure and weakened the role of RDN in mitigating gut microbiota aberrations by impairing intestinal barrier function, resulting in an increase in intestinal bacteria harmful to cardiac function and a decrease in beneficial bacteria in HF rats following RDN.

## MATERIALS AND METHODS

### Animal grouping, TAC, and RDN.

**FIG 11:**
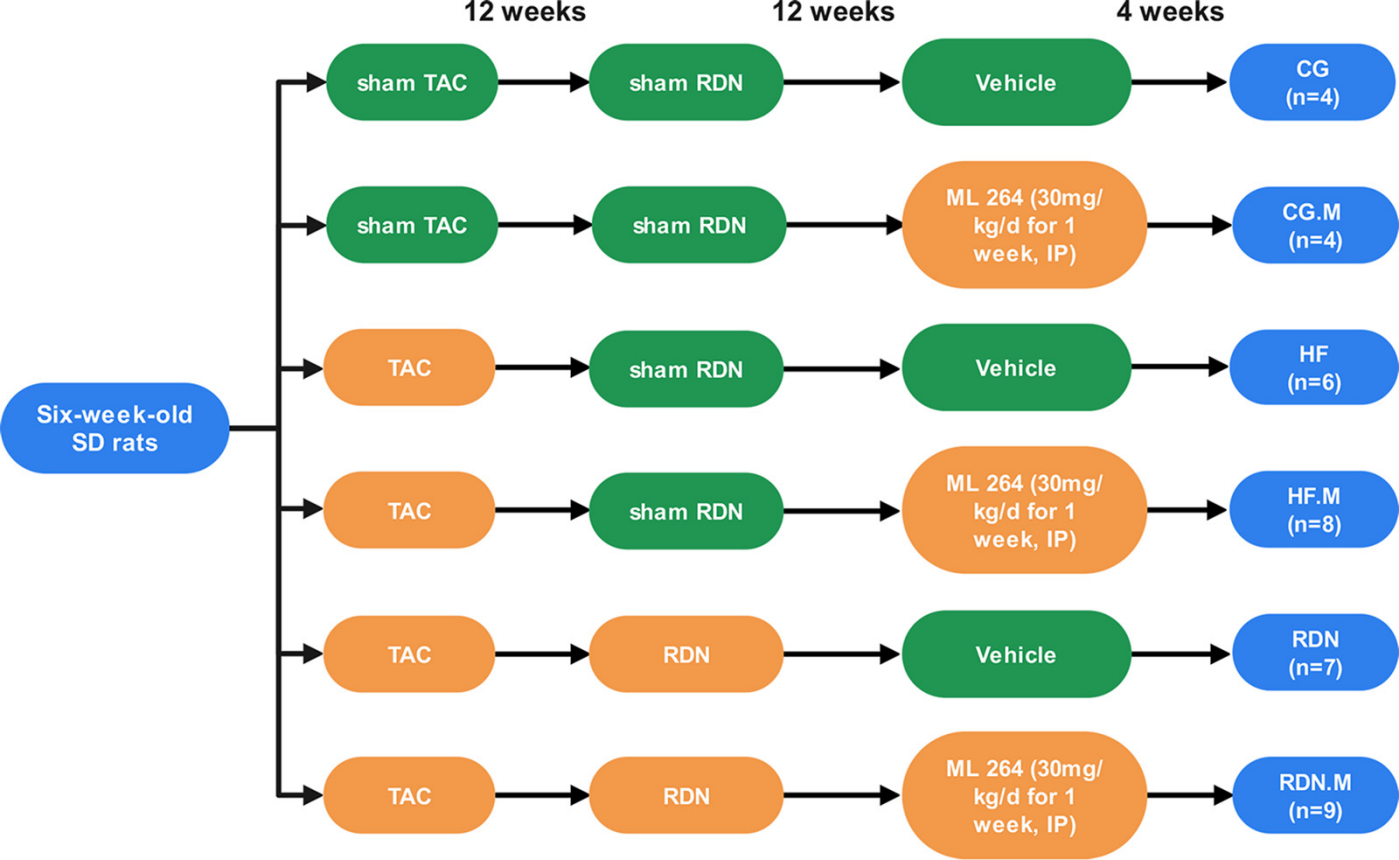
The animal grouping and timeline of the experimental protocol. TAC, transverse aortic constriction; RDN, renal denervation; HF, heart failure.

Sprague-Dawley rats were randomly distributed into six groups as follows: (i) the CG group (*n* = 4), which was the control group; rats received sham TAC, sham RDN, and an intraperitoneal injection of 0.9% saline; (ii) the CG.M group (*n* = 4); rats received sham TAC, sham RDN, and an intraperitoneal injection of ML264 (30 mg/kg of body weight/day), a specific inhibitor of KLF5, for 1 week; (iii) the HF group (*n* = 6); rats received TAC to induce HF, sham RDN, and an intraperitoneal injection of 0.9% saline; (iv) the HF.M group (*n* = 8); rats received TAC, sham RDN, and an intraperitoneal injection of ML264 (30 mg/kg/day) for 1 week; (v) the RDN group (*n* = 7); rats received TAC, RDN, and an intraperitoneal injection of 0.9% saline; (vi) the RDN.M group (*n* = 9); rats received TAC, RDN, and an intraperitoneal injection of ML264 (30 mg/kg/day) for 1 week (Fig. 11).

The TAC, sham TAC, RDN, and sham RDN procedures were performed as described previously (13, 45). Briefly, after anesthesia with 2% pentobarbital sodium (30 mg/kg, intraperitoneally injected), 6-week-old Sprague-Dawley rats, weighing 110 to 200 g, underwent TAC surgery by constricting the abdominal aorta above the left renal arteries. RDN was performed by painting the surface of the left and right renal arteries with phenol (10% phenol in 95% alcohol) at the 12th week after TAC. ML264 administration was conducted at the 12th week after RDN. All rats were euthanized by an overdose of pentobarbital sodium (100 mg/kg) at 34 weeks of age, and jejunum and fecal contents were collected. The investigation conforms to the Guide for the Care and Use of Laboratory Animals published by the U.S. National Institutes of Health. The protocol was approved by the institutional ethics committee of Guangzhou First People’s Hospital.

### Histopathologic examination.

For light microscopy, the jejunal samples were fixed with 4% buffered formaldehyde for 48 h at room temperature and then embedded in paraffin. Paraffin-embedded jejunum sections were prepared, and hematoxylin and eosin (HE) staining was conducted according to routine protocols. Immunohistochemistry of KLF5 (1:500; OriGene, USA) was performed as we previously described (15). The protein expression of KLF5 in the jejunum was assessed by immunohistochemistry and semiquantitative analysis, indicated as the values of the integrated optical density (IOD) measured using the ImageJ pro plus 6.0 image analysis program by a blinded investigator, and the mean IOD was calculated from IOD/area (integrated optical density/positive areas).

The ultrastructure of the intestinal epithelial cells and their connections was observed using transmission electron microscopy (TEM). The cells were fixed with 2.5% glutaraldehyde at 4°C overnight, postfixed in 1% osmium tetroxide for 2 h, dehydrated in a graded series of ethanol baths, and then embedded in Epon 812. Ultrathin sections were stained with uranyl acetate and lead citrate and observed using a JEM-1400 PLUS (Japan Electron Optics Laboratory Co., Ltd). The microvillus length, density, and ratio of length to width were calculated using the ImageJ Pro Plus 6.0 image analysis program.

### Quantitative reverse transcription-PCR.

Total RNA was extracted from jejunum tissues using NucleoZOL reagent (740404.200; Macherey-Nagel, Düren, Germany), and cDNA was synthesized using a reverse transcriptase kit (TaKaRa, Japan). Real-time PCR (RT-PCR) was performed using a SYBR green RT-PCR kit (AG 11701; AgBio, China). Primers were designed to detect KLF5 (forward, CCAAGTCAGTTTCTTCCACAAC; reverse, GTTTCTCCAAATCGGGGTTACT) and desmoglein 2 (DSG2) (forward, AGACCCTAGCCGAAGTTTGC; reverse, TCTGAGCTGGCTGTCACTTG) gene expression based on the sequences available in the NCBI database. The relative mRNA expression was normalized to GAPDH and calculated using the 2^−ΔΔ^*^CT^* formula.

### Western blots.

Jejunum tissues were lysed with radioimmunoprecipitation assay (RIPA) buffer (Beyotime, China). Proteins were electrophoretically separated on SDS-PAGE gels and then transferred onto polyvinylidene difluoride (PVDF) membranes (Millipore, USA). After blocking with 5% nonfat milk, the membranes were incubated with anti-KLF5 (1:500; Origene, USA) or anti-DSG2 (1:1,000; Thermo Fisher, USA) primary antibodies overnight at 4°C. After washing, the specific blots were incubated with the species-appropriate secondary antibodies HRP-conjugated AffiniPure goat anti-mouse IgG for KLF5 (1:5,000; Boster, China) and HRP-conjugated AffiniPure goat anti-rabbit IgG for DSG2 and GAPDH (1:5,000; Boster, China) for 1 h at room temperature. Finally, the protein bands were viewed with an Imaging System ChemiDoc MP (Bio-Rad, USA) and then analyzed with ImageJ software. In the quantitative analysis of Western blots, all of the bands detected were within the linear range of detection.

### Sequencing of 16S rRNA gene.

Fecal samples were collected from the colons of rats with a sterile device, frozen in liquid nitrogen, and then stored at −80°C until further processing. Fecal DNA extraction and sequencing of the 16S rRNA gene were performed as previously described (13). All effective tags of all samples were clustered into operational taxonomic units (OTUs) with 97% identity. Species annotation was performed based on the small subunit rRNA (SSU rRNA) database, and the community composition of each sample at different taxonomic levels was counted. Differences between groups were analyzed by *t* test, and differences were considered statistically significant at a *P* value of <0.05. Beta diversity analysis was used to evaluate differences in species complexity. Beta diversity on unweighted UniFrac distance was calculated by QIIME software (version 1.9.1), and UPGMA sample cluster trees were constructed. Functional classification schemes of KEGG (Kyoto Encyclopedia of Genes and Genomes) orthology based upon bacterial 16S sequencing data were predicted by Tax4Fun.

### Statistical analyses.

Continuous normally distributed variables are expressed as the mean ± standard deviation. Differences between the groups were evaluated by *t* test or Kruskal-Wallis H test. *P* values were two-sided and considered significant when <0.05. Analyses were conducted using R, version 3.5.3.

### Data availability.

The data have been deposited in the database of the National Center for Biotechnology Information (NCBI) under BioProject accession number PRJNA838923.
